# Stroke after lung transplantation: a systematic review and meta-analysis

**DOI:** 10.3389/fmed.2026.1795510

**Published:** 2026-03-31

**Authors:** Mengshan Xie, Fei Zeng, Peipei Gu, Jiangshuyuan Liang, Fangfang Hao, Yandie Wang

**Affiliations:** Department of Nursing, The Second Affiliated Hospital of Zhejiang University School of Medicine, Hangzhou, China

**Keywords:** cerebrovascular accident, extracorporeal membrane oxygenation, incidence, lung transplantation, meta-analysis, mortality, stroke, systematic review

## Abstract

**Background:**

Stroke represents a significant but understudied complication following lung transplantation. No systematic review has synthesized the evidence on post-transplant stroke incidence and outcomes. This study aimed to determine the pooled incidence of stroke after lung transplantation, explore sources of heterogeneity, and synthesize evidence on mortality impact and risk factors.

**Methods:**

We searched PubMed, Embase, Cochrane Library, and Web of Science from inception to December 2025. Studies reporting stroke incidence in adult lung transplant recipients with extractable numerator and denominator data were included. Random-effects meta-analysis was performed for incidence pooling; mortality and risk factor data were synthesized narratively.

**Results:**

Seventeen studies (33,175 patients) were included. The pooled stroke incidence was 3.58% (95% CI: 2.86%–4.48%), with substantial heterogeneity (I^2^ = 69.0%; 95% prediction interval: 1.66%–7.54%). Heterogeneity was not explained by geographic region or time window, but was eliminated after exclusion of the single registry study (I^2^ = 0%), indicating that variation in ascertainment methods was the primary source of between-study variability. Exploratory meta-regression found no association between bilateral transplant proportion and stroke incidence (*p* = 0.86). Sensitivity analyses yielded consistent results. Two studies reported mortality outcomes: stroke was associated with a 3-fold increase in mortality (adjusted hazard ratio 3.01; 95% CI: 2.67–3.41) and markedly higher 30-day mortality (20% vs. 1.3%). In the largest registry study, multivariable analysis identified postoperative extracorporeal membrane oxygenation (ECMO; adjusted odds ratio 2.98), bilateral transplant (adjusted odds ratio 1.71), low-volume center, and older age as risk factors.

**Conclusion:**

Stroke affects approximately 1 in 28 lung transplant recipients and is associated with 3-fold increased mortality. Standardized definitions and prospective studies are needed to address gaps in risk stratification and prevention.

**Systematic review registration:**

https://www.crd.york.ac.uk/prospero/display_record.php?RecordID=1236324, identifier [CRD420251236324].

## Introduction

Lung transplantation is an established life-saving intervention for end-stage lung disease, with over 4,600 procedures performed annually worldwide (1). According to the Scientific Registry of Transplant Recipients, 1-year survival reached 88.5%, and 5-year survival was approximately 60% (2). Despite these improvements, the early postoperative period remains marked by substantial morbidity, with neurological complications occurring in up to 47% of recipients and representing significant contributors to poor outcomes (3).

Among neurological complications, stroke stands out for the severity of its consequences. Although relatively uncommon, it has emerged as a major determinant of outcomes after transplantation. Evidence from institutional cohorts and registry analyses consistently indicates substantially elevated mortality, with a 3-fold increase in adjusted risk compared to those without stroke (4). This mortality burden exceeds that observed after heart transplantation, where stroke confers a nearly 2-fold increased risk of death (5). Beyond mortality, stroke markedly prolongs hospitalization and may cause persistent neurological deficits (6). Collectively, these data establish stroke as a leading contributor to adverse outcomes following lung transplantation.

The pathophysiology of stroke following lung transplantation is multifactorial, involving intraoperative embolism, postoperative atrial fibrillation, and complications of mechanical circulatory support (7). However, reported incidence varies widely across studies (8, 9). Existing evidence derives predominantly from small, single-center retrospective analyses in which stroke definitions and observation windows remain unstandardized. Large multicenter investigations are absent, stroke subtypes are rarely distinguished, and risk factor analyses vary considerably in covariates and analytical approaches, which limits direct comparison across studies. No systematic review has synthesized these fragmented data to provide pooled incidence estimates or identify consistent patterns. We therefore aimed to determine the pooled incidence of stroke after lung transplantation, explore sources of heterogeneity through subgroup and sensitivity analyses, and synthesize available evidence on mortality impact and risk factors.

## Methods

### Protocol and registration

This systematic review was prospectively registered with PROSPERO (CRD420251236324) and conducted following the Preferred Reporting Items for Systematic Reviews and Meta-Analyses (PRISMA) 2020 guidelines (10).

### Search strategy

We systematically searched PubMed/MEDLINE, Embase (via Ovid), Cochrane Library (CENTRAL), and Web of Science Core Collection from inception to 30 December 2025. The search strategy combined MeSH terms and free-text keywords related to “lung transplantation,” “stroke,” “cerebrovascular accident,” and “brain infarction,” with no language restrictions applied during the search phase. The complete search strategy is provided in Supplementary Table 1. Reference lists of included studies were manually screened, and forward citation searches were conducted using Google Scholar.

### Eligibility criteria

Inclusion criteria: (1) Observational studies of adult patients (≥18 years) undergoing lung transplantation; (2) reported stroke incidence with extractable numerator and denominator data; (3) sample size ≥ 20 patients, to ensure minimum statistical reliability for incidence estimation; (4) peer-reviewed original research published in English.

Exclusion criteria: (1) Case reports, case series < 20 patients, reviews, editorials, letters, or conference abstracts; (2) studies reporting only composite neurological outcomes (e.g., encephalopathy, seizures) without stroke-specific data; (3) heart-lung transplant unless lung-only data were separately reported. Studies reporting stroke combined with transient ischemic attack were included but addressed in sensitivity analysis.

### Study selection and data extraction

Records identified from database searches were imported into EndNote and duplicates were removed. Two reviewers independently screened titles and abstracts, followed by full-text assessment of potentially eligible studies. Inter-rater agreement for title and abstract screening was 98.4% (Cohen’s κ = 0.85). Disagreements were resolved through discussion or consultation with a third reviewer.

Data extraction was performed independently by two reviewers using a standardized form. Extracted data included study characteristics (first author, publication year, country, study design, data source, study period, sample size), patient demographics (age, sex, transplant indication), stroke outcomes (number of events, denominator, time window, stroke definition, ascertainment method, subtype if reported), and when available, mortality data and risk factors. For data extraction, stroke was defined as any acute cerebrovascular event reported as stroke, CVA, or cerebral infarction, including ischemic, hemorrhagic, and unclassified subtypes. Ascertainment methods were categorized as objective (neuroimaging or ICD coding) or other (clinical documentation or unspecified). One study reporting stroke combined with TIA as an inseparable composite was included, with its impact assessed through sensitivity analysis. Inter-rater agreement on primary outcome data extraction was 94.1%. When multiple publications reported on potentially overlapping cohorts (e.g., same institution with overlapping study periods), we recorded the overlap. For the primary analysis, we prioritized the study with the largest sample size or most complete outcome reporting.

### Quality assessment

Methodological quality was assessed using the Newcastle-Ottawa Scale (NOS) adapted for incidence studies (11), with emphasis on sample representativeness and outcome ascertainment rather than comparative analysis. The scale evaluates three domains: selection (maximum four stars), comparability (maximum two stars), and outcome (maximum three stars), with a total possible score of nine stars. Total scores of 7–9 were considered high quality, 4–6 moderate quality, and 0–3 low quality. Quality assessment was performed independently by two reviewers. The intra-class correlation coefficient for total NOS scores was 0.72, indicating good inter-rater agreement. Disagreements were resolved by consultation with a third reviewer. Detailed quality scores are provided in Supplementary Table 2.

### Statistical analysis

Statistical analyses were performed using R (version 4.4) with the “meta” package (version 7.0). Pooled stroke incidence was calculated using random-effects meta-analysis with logit transformation and the DerSimonian-Laird estimator for between-study variance (τ^2^). Heterogeneity was assessed using Cochran’s Q test and the I^2^ statistic, with values of 25%, 50%, and 75% representing low, moderate, and substantial heterogeneity, respectively. Prediction intervals were calculated to estimate the expected range of true incidence across settings.

Subgroup analyses were prespecified by geographic region (United States vs. non-United States) and time window (early postoperative vs. extended follow-up). Additional subgroup analyses were performed by study design (registry vs. single-center) and stroke ascertainment method (objective vs. other). An exploratory meta-regression was conducted with the study-level proportion of bilateral transplants as a continuous moderator to assess whether it explained between-study heterogeneity in stroke incidence. Sensitivity analyses included leave-one-out analysis, exclusion of the large registry study (4) to assess potential double-counting with overlapping US single-center cohorts, exclusion of studies including transient ischemic attack (12), and restriction to studies with objective stroke confirmation (imaging or ICD-coded). Publication bias was assessed using funnel plot inspection, Egger’s regression test, and Peters’ test, with trim-and-fill analysis performed for reference.

For stroke incidence, quantitative meta-analysis was performed. For mortality and risk factors, we prespecified that quantitative synthesis required ≥3 studies with comparable effect measures. As only two studies reported these outcomes with heterogeneous measures and follow-up periods, narrative synthesis was performed. Studies excluded from the primary incidence analysis due to overlapping populations or incompatible time windows were considered for narrative synthesis of mortality and risk factor data where they met other eligibility criteria and reported relevant outcomes.

## Results

### Study selection

**FIGURE 1:**
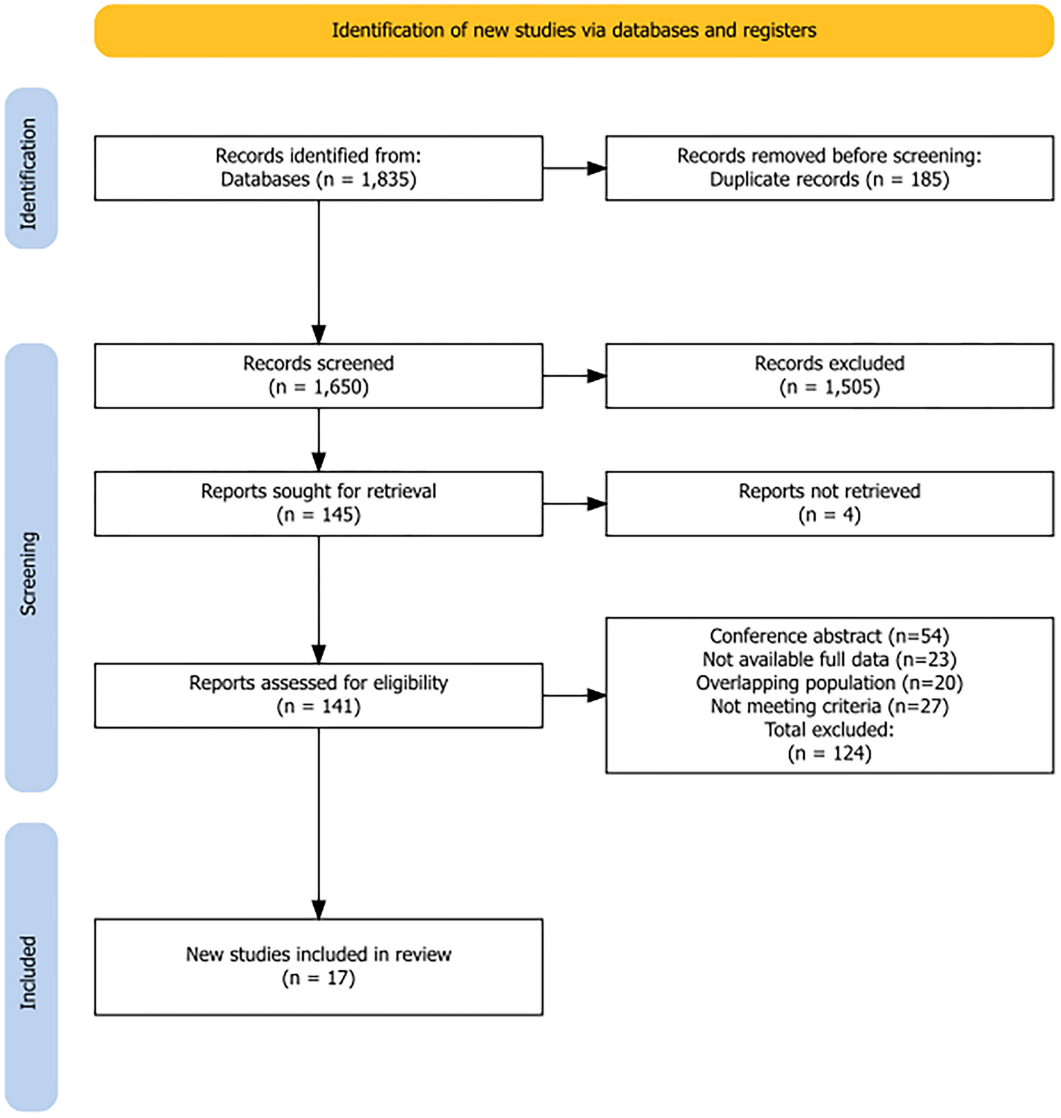
PRISMA flow diagram of study selection. Flow diagram illustrating the systematic literature search and study selection process following PRISMA 2020 guidelines. A total of 1,835 records were identified from electronic databases (PubMed, *n* = 257; Embase, *n* = 1,132; Web of Science, *n* = 428; Cochrane Library, *n* = 18). After removing 185 duplicates, 1,650 records underwent title and abstract screening, of which 1,505 were excluded. Of the 145 reports sought for retrieval, four could not be retrieved and 141 full-text articles were assessed for eligibility. A total of 124 reports were excluded for the following reasons: conference abstracts (*n* = 54), no extractable stroke data (*n* = 23), overlapping population (*n* = 20), and other reasons (*n* = 27). Seventeen studies met inclusion criteria for the systematic review and meta-analysis.

The systematic search across electronic databases initially identified 1,835 records (PubMed 257, Embase 1,132, Cochrane 18, Web of Science 428). After removing 185 duplicates, 1,650 records remained for title and abstract screening. Manual screening of reference lists and forward citation searches identified no additional eligible studies. Following initial screening, 145 articles were selected for full-text review, of which four could not be retrieved. After excluding 124 articles for reasons including conference abstracts, lack of extractable stroke data, and overlapping populations, 17 studies were ultimately included in the final analysis (Figure 1).

### Study characteristics

The 17 included studies, published between 2006 and 2024, comprised 33,175 lung transplant recipients from six countries (Table 1). Ten studies originated from the United States (4, 12–20), six from Europe (3, 6, 9, 21–23), and one from Japan (8). All were retrospective cohorts with sample sizes ranging from 28 to 28,564 patients. One study (4) utilized the United Network for Organ Sharing (UNOS) national registry (*n* = 28,564; 86.1% of total sample), while 16 were single-center cohorts. Study periods spanned 1995–2021. Eleven studies (3, 4, 6, 8, 12–14, 16–19) reported stroke events during the early postoperative period or index hospitalization, while four studies used unspecified timeframes (9, 15, 22, 23), and two reported overall follow-up extending beyond the early postoperative period (20, 21). For subgroup analysis by time window, the four studies with unspecified timeframes were excluded due to inability to classify definitively. Three studies (13, 14, 21) reported stroke subtypes, with ischemic events predominating (72%–95%). Stroke ascertainment methods varied considerably, with four studies using objective confirmation through imaging or ICD coding (3, 13, 14, 21), seven relying on clinical documentation or registry coding (4, 6, 8, 12, 17, 20, 22), and six not specifying the method (9, 15, 16, 18, 19, 23). One study (12) combined stroke and transient ischemic attack as a composite outcome. Detailed characteristics of the selected studies are presented in Table 1.

**TABLE 1:** Characteristics of included studies.

References	Year	Country	Design	Sample size	Stroke events	Incidence (%)	Time window	Ascertainment	NOS
Shou et al. (4)	2023	USA	Registry	28,564	653	2.3	In-hospital	Registry code	9
Shigemura et al. (13)	2013	USA	SC	759	29	3.8	≤2 weeks	Imaging	6
Chan et al. (15)	2023	USA	SC	557	18	3.2	Postop*	Unclear	6
Zanotti et al. (16)	2014	USA	SC	539	20	3.7	≤30 days	Unclear	7
Marin-Diez et al. (21)	2021	Spain	SC	344	17	4.9	Overall	Imaging	7
Makey et al. (17)	2018	USA	SC	274	6	2.2	≤30 days	Clinical	6
Siddiqui and Shakil (18)	2024	USA	SC	232	7	3.0	≤30 days	Unclear	6
Moneke et al. (6)	2023	Germany	SC	221	13	5.9	≤1 month	Clinical	9
Smith et al. (19)	2006	USA	SC	182	7	3.8	In-hospital	Unclear	7
Kalsbeek et al. (14)	2022	USA	SC	476	20	4.2	≤36 days	Imaging	7
Akagi et al. (8)	2017	Japan	SC	28	2	7.1	≤30 days	Clinical	7
Mohite et al. (9)	2014	UK	SC	184	0	0	Postop*	Unclear	9
Bates et al. (20)	2017	USA	SC	79	3	3.8	Overall	Clinical	6
Smith et al. (12)	2018	USA	SC	276	11	4.0	≤7 days	Clinical	9
Orlitova (22)	2023	Belgium	SC	156	3	1.9	Periop	Clinical	6
Lusebrink et al. (23)	2024	Germany	SC	196	8	4.1	Overall*	Unclear	8
Gamez et al. (3)	2017	Spain	SC	108	5	4.6	≤2 months	ICD-10	9

SC, single-center; Registry, national/multicenter registry study; NOS, Newcastle-Ottawa Scale score (maximum 9); Postop, postoperative; Periop, perioperative; Unclear, ascertainment method not specified in original publication. *Time window not explicitly defined.

### Quality assessment

Methodological quality was assessed using the Newcastle-Ottawa Scale (Supplementary Table 2). Total scores ranged from 6 to 9 stars (median 7), with eleven studies (64.7%) rated high quality (≥7 stars) and six (35.3%) moderate quality (4–6 stars); no study scored below 4. In the selection domain, six studies (8, 15–18, 22) enrolled selected subgroups rather than representative cohorts, including specific surgical populations, disease subsets, or patients meeting particular clinical criteria; all studies clearly ascertained transplant status. For incidence studies without a comparison group, the comparability domain was interpreted as whether studies accounted for potential confounders when reporting risk factors. Nine studies (13–15, 17–22) did not perform multivariable adjustment for stroke-specific risk factors, primarily due to low event numbers precluding regression modeling; two studies (8, 16) achieved partial adjustment through limited multivariable models or baseline matching; and six studies (3, 4, 6, 9, 12, 23) achieved full adjustment through multivariable analysis or propensity matching. In the outcome domain, all studies used appropriate methods for stroke ascertainment, though definitions varied from imaging confirmation to clinical documentation; follow-up duration was adequate for early stroke detection in most studies, with one study (20) having >20% attrition. No study was excluded based on quality assessment.

### Meta-analysis of stroke incidence

**FIGURE 2:**
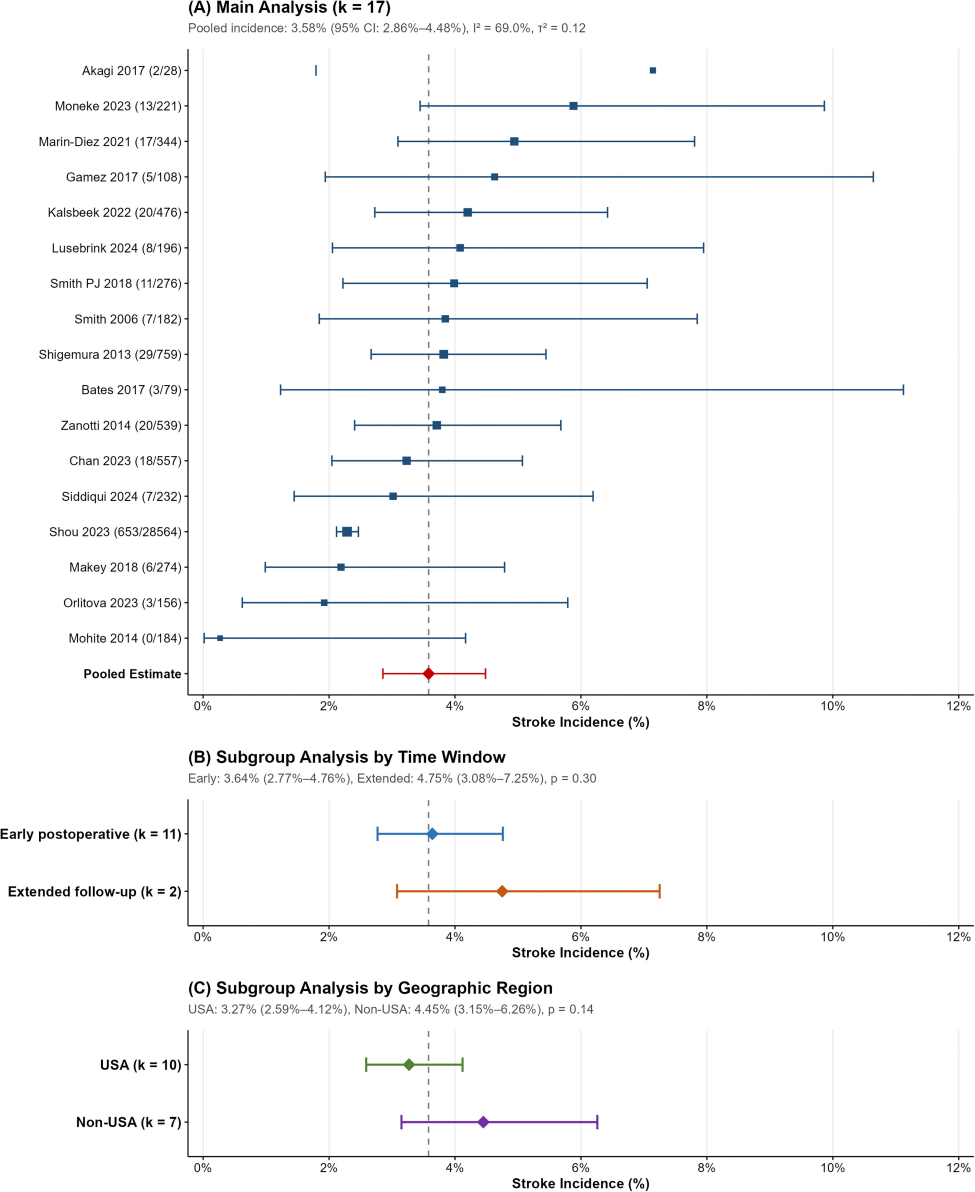
Forest plots of stroke incidence after lung transplantation. **(A)** Main analysis: random-effects meta-analysis of stroke incidence across 17 studies comprising 33,175 lung transplant recipients. Each horizontal line represents a study with its point estimate (square) and 95% confidence interval. Square size is proportional to study weight. The diamond represents the pooled incidence estimate (3.58%; 95% CI: 2.86%–4.48%). Heterogeneity statistics: I^2^ = 69.0%, τ^2^ = 0.12, Q test *p* < 0.001. The 95% prediction interval (1.66%–7.54%) indicates the expected range of true incidence in future comparable settings. **(B)** Subgroup analysis by time window: studies reporting early postoperative stroke (*k* = 11) showed pooled incidence of 3.64% (95% CI: 2.77%–4.76%), while studies with extended follow-up (*k* = 2) showed 4.75% (95% CI: 3.08%–7.25%). Test for subgroup difference: *p* = 0.30. **(C)** Subgroup analysis by geographic region: studies from the United States (*k* = 10) showed pooled incidence of 3.27% (95% CI: 2.59%–4.12%), while non-United States studies (*k* = 7) showed 4.45% (95% CI: 3.15%–6.26%). Test for subgroup difference: *p* = 0.14.

Seventeen studies comprising 33,175 lung transplant recipients reported stroke incidence ranging from 0% to 7.1%. Heterogeneity testing revealed an I^2^ statistic of 69.0% (τ^2^ = 0.12; Q test *p* < 0.001), and a random-effects model was used for the meta-analysis. The forest plot in Figure 2 illustrates the incidence rates and their corresponding confidence intervals. The pooled stroke incidence was 3.58% (95% CI: 2.86%–4.48%), with a total of 822 events. The 95% prediction interval of 1.66%–7.54% indicates the expected range of true incidence across clinical settings.

#### Subgroup analyses

Prespecified subgroup analyses revealed no significant differences by geographic region (United States 3.27%, *k* = 10 vs. non-United States 4.45%, *k* = 7; *p* = 0.14) or time window (early postoperative 3.64%, *k* = 11 vs. extended follow-up 4.75%, *k* = 2; *p* = 0.30), with detailed estimates in Table 2. The numerically higher incidence in non-United States studies and with extended follow-up is consistent with regional practice variations and cumulative stroke risk over time.

**TABLE 2:** Summary of subgroup and sensitivity analyses.

Analysis	*k*	Pooled incidence (95% CI)	I^2^	Interpretation
Main analysis	17	3.58% (2.86%–4.48%)	69.0%	Reference estimate
Subgroup by region				
USA	10	3.27% (2.59%–4.12%)	64.1%	P for interaction = 0.14
Non-USA	7	4.45% (3.15%–6.26%)	24.6%	–
Subgroup by time window				
Early postoperative	11	3.64% (2.77%–4.76%)	73.3%	P for interaction = 0.30
Extended follow-up	2	4.75% (3.08%–7.25%)	0%	Four studies excluded
Subgroup by ascertainment				
Objective (imaging/ICD)	4	4.23% (3.36%–5.30%)	0%	P for interaction = 0.16
Other	13	3.31% (2.56%–4.27%)	72.0%	–
Meta-regression				
Bilateral transplant proportion	15	–	–	Coefficient = −0.0012; R^2^ = 0%; *p* = 0.86
Sensitivity analyses				
Excluding Shou 2023 (registry)	16	3.89% (3.35%–4.50%)	0%	Heterogeneity eliminated
Excluding Smith_PJ 2018 (TIA)	16	3.56% (2.81%–4.49%)	69.6%	Minimal change
Objective confirmation only	4	4.23% (3.36%–5.30%)	0%	Same subset as ascertainment subgroup above

Additional subgroup analysis by stroke ascertainment method showed no significant difference between objective confirmation and other methods (4.23%, *k* = 4 vs. 3.31%, *k* = 13; *p* = 0.16). Formal subgroup comparison by study design was not feasible as only one registry-based study was included. Its exclusion is addressed in the sensitivity analysis below. Exploratory meta-regression with the study-level proportion of bilateral transplants as a moderator showed no significant association with stroke incidence (coefficient = −0.0012; *p* = 0.86; *k* = 15).

#### Sensitivity analyses

Multiple sensitivity analyses confirmed the robustness of the main estimate (Table 2). Leave-one-out analysis showed stable pooled incidence (range 3.44%–3.89%) (Supplementary Figure 1), and excluding the TIA-inclusive study (12) yielded virtually unchanged results (3.56%; 95% CI: 2.81%–4.49%). Two analyses provided insight into heterogeneity sources: excluding the large registry study (4) eliminated heterogeneity (I^2^ from 69.0% to 0%), with the 16 single-center studies showing pooled incidence of 3.89% (95% CI: 3.35%–4.50%) (Supplementary Figure 2); similarly, restricting to objectively confirmed cases (imaging or ICD coding; *k* = 4) yielded homogeneous results (I^2^ = 0%; pooled incidence 4.23%; 95% CI: 3.36%–5.30%) (Supplementary Figure 4). These findings indicate that variation in ascertainment methods—rather than study design—drives between-study heterogeneity.

#### Publication bias

**FIGURE 3:**
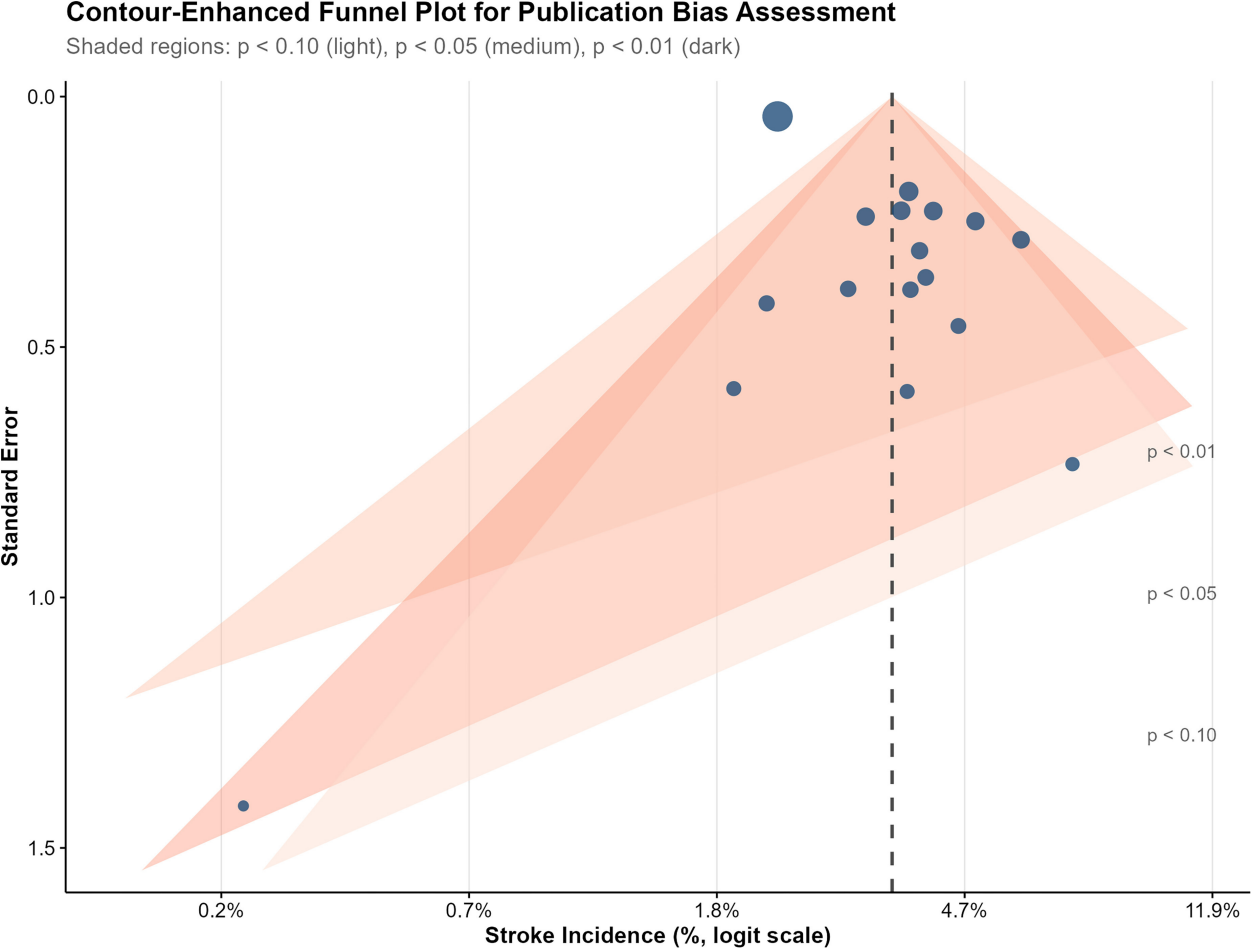
Contour-enhanced funnel plot for assessment of publication bias. Contour-enhanced funnel plot displaying the relationship between study precision (standard error of logit-transformed incidence, y-axis) and effect size (logit incidence, x-axis). Each point represents an individual study. The vertical dashed line indicates the pooled estimate. Shaded contours represent regions of statistical significance (*p* < 0.01, *p* < 0.05, *p* < 0.10), allowing visual assessment of whether asymmetry reflects publication bias or other sources. Visual inspection shows mild asymmetry, which likely reflects heterogeneity in stroke ascertainment methods rather than true publication bias.

Funnel plot inspection revealed mild asymmetry (Figure 3), with Egger’s regression test (*p* = 0.003) and Peters’ test (*p* = 0.007) indicating small-study effects. Trim-and-fill analysis did not impute any missing studies (Supplementary Figure 3). However, stable estimates across sensitivity analyses and elimination of heterogeneity (I^2^ = 0%) when restricting to objectively confirmed cases suggest this asymmetry reflects ascertainment heterogeneity rather than true publication bias.

### Outcomes associated with stroke

Stroke following lung transplantation carries substantial prognostic implications. Two included studies (4, 14) provided comparative mortality data, both demonstrating markedly worse outcomes in affected patients. In the UNOS registry analysis encompassing 28,564 recipients, Shou et al. (4) observed progressively diverging survival curves: mortality differences between stroke and non-stroke patients widened from 14% at 1 month to 28% at 24 months, and stroke remained independently associated with a 3-fold increase in mortality after adjustment for 20 covariates (aHR = 3.01; 95% CI: 2.67–3.41). Kalsbeek et al. (14) reported similar findings in a single-center cohort, with 30-day mortality reaching 20% among stroke patients compared with 1.3% in those unaffected (*p* < 0.001); this survival disadvantage persisted over long-term follow-up (log-rank *p* = 0.044). Courtwright et al. (24), in a registry study excluded from our incidence analysis due to population overlap, identified stroke as the strongest predictor of hospital mortality (aOR = 4.49; 95% CI: 3.01–6.68)—one in four stroke patients died during the index hospitalization. Stroke also nearly doubled the median length of stay (30 vs. 16 days, *p* < 0.001) (4), with most events occurring within the first two postoperative weeks (median onset 7–16 days) (3, 14). Limited data suggest stroke survivors may experience residual neurological deficits (6).

### Risk factors for stroke

Two included studies (4, 14) examined predictors of stroke, revealing that procedure-related factors outweighed traditional cardiovascular risk factors. In the UNOS registry analysis, Shou et al. (4) identified postoperative ECMO as the strongest predictor (aOR = 2.98; 95% CI: 2.19–4.06), followed by bilateral transplantation (aOR = 1.71; 95% CI: 1.36–2.15) and prolonged ischemia time (aOR = 1.07 per hour; 95% CI: 1.03–1.12). Recipient characteristics including older age, worse functional status, lower BMI, and elevated creatinine conferred more modest risk elevations (aOR range 1.07–1.13). Management at low-volume centers also emerged as a significant factor (aOR = 1.37; 95% CI: 1.06–1.79), whereas diabetes and tobacco use showed no association. Bilateral transplantation was also associated with higher stroke incidence (2.6% vs. 1.5%) (4). In a smaller single-center cohort, Kalsbeek et al. (14) found higher lung allocation score to be the only significant predictor (median 46.2 vs. 37.4, *p* = 0.003), though limited events (*n* = 20) precluded multivariable analysis. Registry studies excluded from our incidence analysis due to population overlap provide additional insights: prolonged ischemia (≥6 h) was associated with elevated incidence (2.6% vs. 1.8%, *p* = 0.01) (25), and connective tissue disease-associated interstitial lung disease emerged as an independent predictor (aOR = 1.75; 95% CI: 1.12–2.74) (26).

## Discussion

### Summary of main findings

To our knowledge, this systematic review represents the first synthesis of evidence on stroke following lung transplantation. Pooling data from 17 studies, we found that approximately 1 in 28 recipients experience stroke, with considerable variability across centers as reflected by the wide prediction interval. Despite limited mortality data, stroke consistently emerged as a major determinant of outcomes, tripling adjusted mortality risk (4) and ranking among the strongest predictors of hospital death (24). The stroke incidence we observed is similar to that reported after cardiac transplantation (27), but exceeds rates in kidney transplant recipients (28), likely reflecting the hemodynamic demands of thoracic surgery, including cardiopulmonary bypass exposure and the frequent need for postoperative mechanical circulatory support. The pooled estimate of 3.58% provides an evidence-based benchmark for patient counseling, while the wide prediction interval (1.66%–7.54%) emphasizes the need for discussing center-specific outcomes. Patients receiving postoperative ECMO (aOR = 2.98), undergoing bilateral transplant (aOR = 1.71), or managed at low-volume centers (aOR = 1.37) represent identifiable high-risk subgroups warranting heightened vigilance.

### Sources of heterogeneity

The substantial heterogeneity observed across studies appears to reflect methodological variability rather than true differences in stroke risk. Stroke ascertainment emerged as the most influential factor: studies using imaging confirmation yielded homogeneous results, whereas those relying on clinical documentation reported lower and more variable rates. This disparity likely reflects the inherent difficulty of stroke recognition in the postoperative setting, where sedation, encephalopathy, and delirium can mask focal neurological deficits (29). Without systematic imaging, subtle events are easily overlooked or misattributed to metabolic causes.

Our pooled data support these associations, though the mechanistic interpretations below remain speculative. Variation in time window definitions introduced additional complexity with important mechanistic implications. Strokes in the immediate postoperative period likely arise from intraoperative embolic events, including manipulation of the left atrium and pulmonary veins, air embolism, and atheroembolism from aortic instrumentation (30). Later events may instead reflect atrial fibrillation-related thromboembolism. The left atrial cuff anastomosis disrupts normal atrial architecture and creates an arrhythmogenic substrate (7), while anticoagulation is often deferred due to bleeding concerns (31). Because atrial fibrillation peaks during the first postoperative week (32), studies with extended follow-up would capture these later events, potentially explaining their higher incidence estimates, although our subgroup analysis (*k* = 2) lacked statistical power to confirm this hypothesis. Exclusion of the large UNOS registry study eliminated heterogeneity entirely, suggesting that administrative databases may detect clinically mild events that single-center reviews focused on major complications might miss. Shou et al. additionally reported an increasing temporal trend in stroke incidence over the study period (4), which may reflect improved detection capabilities rather than a true increase in stroke risk. These methodological differences demonstrate the need for standardized definitions and prospective ascertainment. In cardiac surgery, similar challenges were addressed through consensus frameworks such as NeuroARC (33). Accordingly, we propose that future lung transplant studies consider: (1) the AHA/ASA tissue-based stroke definition (34), requiring either neuroimaging confirmation or a clinical deficit persisting beyond 24 h; (2) standardizing “early” stroke as occurring within 30 days of transplantation, consistent with established perioperative reporting conventions; and (3) reporting ascertainment method, stroke subtype (ischemic versus hemorrhagic), and TIA events separately. Given that ascertainment method was the primary driver of between-study heterogeneity in our analysis, protocol-driven neurological assessment would facilitate comparison across future studies.

### Risk factors and prognostic implications

The risk factor analysis revealed a striking dominance of procedural factors over traditional cardiovascular risk factors. Postoperative ECMO emerged as the strongest predictor, with an effect size exceeding that of bilateral transplantation, low-volume center management, and prolonged ischemia time. This association likely reflects confounding by indication, as postoperative ECMO is typically initiated for severe primary graft dysfunction with hemodynamic compromise, rather than an isolated ECMO effect. One included study (15) found no difference in stroke incidence across intraoperative support strategies (ECMO, cardiopulmonary bypass, or off-pump), suggesting that risk is specific to postoperative support and consistent with a PGD-ECMO-stroke cascade. The putative mechanistic basis for ECMO-associated stroke involves both thromboembolic events and cerebral hypoperfusion; in VA-ECMO, oxygenated blood returns directly to the aorta without the pulmonary circulation acting as a filter for emboli (35). In the ELSO registry, neurologic complications occurred in 15.1% of VA-ECMO patients (36). Beyond ECMO, several hypothesized embolic pathways merit consideration: bilateral transplantation with dual left atrial anastomoses increases thrombogenic surface (4), post-transplant atrial fibrillation provides a substrate for cardioembolism (7), pulmonary vein thrombosis develops in 15% of recipients within 48 h (37) and has been identified as a potential embolic source in stroke patients (13), and patent foramen ovale may enable paradoxical embolism (38). The absence of associations with diabetes and tobacco use contrasts with stroke epidemiology in the general population, likely reflecting both the dominance of acute procedural factors and selection effects from rigorous pre-transplant cardiovascular evaluation. Studies differentiating stroke subtypes consistently found ischemic events predominating (72%–95%), suggesting embolic mechanisms as a major contributor (13, 14, 21). This embolic predominance supports the role of surgical manipulation, left atrial anastomoses, and early atrial fibrillation in stroke pathogenesis. The potential benefit of PFO closure warrants investigation, as one study reported numerically lower stroke rates after repair (0% vs. 5%), although this difference was not statistically significant (*p* = 0.20) (38). In contrast, all strokes in ECLS-supported patients were hemorrhagic (22), suggesting anticoagulation requirements shift the subtype distribution in this high-risk population.

The prognostic implications of post-transplant stroke are profound. Although limited to two studies, the available data consistently demonstrate that stroke fundamentally alters the clinical trajectory. The 3-fold increase in adjusted mortality risk persisted over extended follow-up (4), with survival curves progressively diverging, suggesting that stroke triggers a cascade of complications rather than a single acute insult. Early mortality was particularly striking, with one in five stroke patients dying within 30 days compared with fewer than 2% of unaffected recipients (14). Beyond mortality, stroke nearly doubled length of stay, with most events occurring within the first two postoperative weeks (3, 4, 14), a period when patients are already at highest risk for other complications. These observations suggest that stroke may serve as a marker of global perioperative injury, capturing patients who have experienced multiple insults including primary graft dysfunction, hemodynamic instability, and prolonged mechanical support. Limited data on functional outcomes suggest substantial morbidity among survivors, with residual neurological deficits reported in stroke patients (6). However, assessment of long-term functional recovery, quality of life, and rehabilitation needs remains an important gap requiring prospective investigation.

This review has several limitations. First, all included studies were observational and predominantly retrospective, with inherent risks of selection bias and unmeasured confounding. Second, significant heterogeneity existed in stroke definitions and ascertainment methods; although sensitivity analyses were conducted, residual heterogeneity remained substantial. Third, limited studies reported mortality or performed multivariable risk factor analysis, restricting quantitative synthesis for these outcomes. Additionally, most studies did not differentiate ischemic from hemorrhagic stroke, limiting mechanistic interpretation. Although no language restrictions were applied during database searching, only English-language publications met our inclusion criteria, which may have excluded relevant data from non-English sources. The exploratory meta-regression examining bilateral transplant proportion was conducted at the study level and is therefore subject to ecological fallacy; the individual-level evidence reported by Shou et al. (4) provides more reliable assessment of this association. Finally, the predominance of US-based studies may limit generalizability to other healthcare settings.

## Conclusion

This systematic review and meta-analysis demonstrates that stroke affects approximately 1 in 28 lung transplant recipients and is associated with 3-fold increased mortality. The substantial heterogeneity observed largely reflects variability in ascertainment methods rather than true differences in stroke risk. Postoperative ECMO and bilateral transplant were identified as key risk factors in the largest registry study, while traditional cardiovascular risk factors showed no significant associations. These findings establish stroke as a significant but understudied complication of lung transplantation. Future research should prioritize consensus on stroke definitions and time windows to facilitate cross-study comparisons, prospective studies with protocol-driven neuroimaging to determine true incidence, and mechanistic investigations of embolic pathways. Given the high burden of post-transplant atrial fibrillation, studies evaluating anticoagulation strategies that balance stroke prevention against bleeding risk are particularly needed.

## Data Availability

The original contributions presented in this study are included in the article/Supplementary materials, further inquiries can be directed to the corresponding author.
