# Supplementary material for: Stroke after lung transplantation: a systematic review and meta-analysis
Source: Front Med (Lausanne). 2026 Mar 31;13:1795510. doi: 10.3389/fmed.2026.1795510 (PMC13076320; doi:10.3389/fmed.2026.1795510)
Supplement: Supplementary file 1 [file Table_1.docx]

**PRISMA 2020 Checklist**

*Stroke After Lung Transplantation: A Systematic Review and Meta-Analysis*

| **Section and Topic** | **Item #** | **Checklist Item** | **Location** |
| --- | --- | --- | --- |
| **TITLE** | 1 | Identify the report as a systematic review. | Title |
| **ABSTRACT** | 2 | See the PRISMA 2020 for Abstracts checklist. | Abstract |
| **INTRODUCTION** |  |  |  |
| Rationale | 3 | Describe the rationale for the review in the context of existing knowledge. | Introduction, para 1-3 |
| Objectives | 4 | Provide an explicit statement of the objective(s) or question(s) the review addresses. | Introduction, para 3 |
| **METHODS** |  |  |  |
| Eligibility criteria | 5 | Specify the inclusion and exclusion criteria for the review and how studies were grouped for the syntheses. | Methods, Eligibility Criteria |
| Information sources | 6 | Specify all databases, registers, websites, organisations, reference lists and other sources searched or consulted to identify studies. | Methods, Search Strategy |
| Search strategy | 7 | Present the full search strategies for all databases, registers and websites, including any filters and limits used. | Supplementary Table 1 |
| Selection process | 8 | Specify the methods used to decide whether a study met the inclusion criteria of the review. | Methods, Study Selection |
| Data collection process | 9 | Specify the methods used to collect data from reports, including how many reviewers collected data from each report. | Methods, Data Extraction |
| Data items | 10a | List and define all outcomes for which data were sought. | Methods, Data Extraction |
|  | 10b | List and define all other variables for which data were sought. | Methods, Data Extraction |
| Study risk of bias assessment | 11 | Specify the methods used to assess risk of bias in the included studies. | Methods, Quality Assessment |
| Effect measures | 12 | Specify for each outcome the effect measure(s) used in the synthesis or presentation of results. | Methods, Statistical Analysis |
| Synthesis methods | 13a | Describe the processes used to decide which studies were eligible for each synthesis. | Methods, Statistical Analysis |
|  | 13b | Describe any methods required to prepare the data for presentation or synthesis. | Methods, Statistical Analysis |
|  | 13c | Describe any methods used to tabulate or visually display results of individual studies and syntheses. | Methods, Statistical Analysis |
|  | 13d | Describe any methods used to synthesize results and provide a rationale for the choice(s). | Methods, Statistical Analysis |
|  | 13e | Describe any methods used to explore possible causes of heterogeneity among study results. | Methods, Statistical Analysis |
|  | 13f | Describe any sensitivity analyses conducted to assess robustness of the synthesized results. | Methods, Statistical Analysis |
| Reporting bias assessment | 14 | Describe any methods used to assess risk of bias due to missing results in a synthesis. | Methods, Statistical Analysis |
| Certainty assessment | 15 | Describe any methods used to assess certainty in the body of evidence for an outcome. | N/A |
| **RESULTS** |  |  |  |
| Study selection | 16a | Describe the results of the search and selection process. | Results, Study Selection; Figure 1 |
|  | 16b | Cite studies that might appear to meet the inclusion criteria, but which were excluded, and explain why they were excluded. | Results, Study Selection |
| Study characteristics | 17 | Cite each included study and present its characteristics. | Results, Study Characteristics; Table 1 |
| Risk of bias in studies | 18 | Present assessments of risk of bias for each included study. | Results, Quality Assessment; Supp Table 2 |
| Results of individual studies | 19 | For all outcomes, present for each study (a) summary statistics for each group and (b) an effect estimate and its precision. | Table 1; Figure 2 |
| Results of syntheses | 20a | For each synthesis, briefly summarise the characteristics and risk of bias among contributing studies. | Results, Meta-Analysis |
|  | 20b | Present results of all statistical syntheses conducted. | Results, Meta-Analysis; Figure 2 |
|  | 20c | Present results of all investigations of possible causes of heterogeneity among study results. | Results, Subgroup Analyses; Table 2 |
|  | 20d | Present results of all sensitivity analyses conducted to assess the robustness of the synthesized results. | Results, Sensitivity Analyses; Table 2 |
| Reporting biases | 21 | Present assessments of risk of bias due to missing results for each synthesis assessed. | Results, Publication Bias; Figure 3 |
| Certainty of evidence | 22 | Present assessments of certainty in the body of evidence for each outcome assessed. | N/A |
| **DISCUSSION** |  |  |  |
| Discussion | 23a | Provide a general interpretation of the results in the context of other evidence. | Discussion, Summary of Main Findings |
|  | 23b | Discuss any limitations of the evidence included in the review. | Discussion, para before Conclusion |
|  | 23c | Discuss any limitations of the review processes used. | Discussion, para before Conclusion |
|  | 23d | Discuss implications of the results for practice, policy, and future research. | Conclusion |
| **OTHER INFORMATION** |  |  |  |
| Registration and protocol | 24a | Provide registration information for the review, including register name and registration number. | Methods, Protocol and Registration |
|  | 24b | Indicate where the review protocol can be accessed, or state that a protocol was not prepared. | Methods, Protocol and Registration |
|  | 24c | Describe and explain any amendments to information provided at registration or in the protocol. | N/A |
| Support | 25 | Describe sources of financial or non-financial support for the review. | Funding |
| Competing interests | 26 | Declare any competing interests of review authors. | Conflict of Interest |
| Availability of data | 27 | Report which of the following are publicly available and where they can be found: template data collection forms; data extracted from included studies; data used for all analyses; analytic code; any other materials used in the review. | Data Availability Statement |

*From: Page MJ, McKenzie JE, Bossuyt PM, et al. The PRISMA 2020 statement: an updated guideline for reporting systematic reviews. BMJ 2021;372:n71. doi: 10.1136/bmj.n71*
