# Supplementary material for: Stroke after lung transplantation: a systematic review and meta-analysis
Source: Front Med (Lausanne). 2026 Mar 31;13:1795510. doi: 10.3389/fmed.2026.1795510 (PMC13076320; doi:10.3389/fmed.2026.1795510)
Supplement: Supplementary file 2 [file Table_2.docx]

**Supplementary Material**

*for: Stroke After Lung Transplantation: A Systematic Review and Meta-Analysis*

**Supplementary Table 1. Search Strategies**

| **Database** | **Search Strategy** |
| --- | --- |
| **PubMed/MEDLINE** | (("Lung Transplantation"[Mesh] OR "lung transplant*"[tiab] OR "pulmonary transplant*"[tiab] OR LuTx[tiab] OR SLTx[tiab] OR BLTx[tiab] OR DLTx[tiab]) AND ("Stroke"[Mesh] OR "Cerebrovascular Disorders"[Mesh] OR "Brain Ischemia"[Mesh] OR "Intracranial Hemorrhages"[Mesh] OR stroke*[tiab] OR cerebrovascular*[tiab] OR "cerebrovascular accident"[tiab] OR CVA[tiab] OR "ischemic stroke"[tiab] OR "hemorrhagic stroke"[tiab] OR "brain infarction"[tiab] OR "cerebral infarct*"[tiab] OR "intracerebral hemorrhage"[tiab] OR "intracranial hemorrhage"[tiab])) NOT (animals[mh] NOT humans[mh]) |
| **Embase (via Ovid)** | 1. exp lung transplantation/ 2. (lung adj3 transplant*).ti,ab. 3. (pulmonary adj3 transplant*).ti,ab. 4. (LuTx or SLTx or BLTx or DLTx).ti,ab. 5. 1 or 2 or 3 or 4 6. exp cerebrovascular accident/ 7. exp brain ischemia/ 8. exp intracranial hemorrhage/ 9. stroke*.ti,ab. 10. cerebrovascular*.ti,ab. 11. (cerebral adj3 infarct*).ti,ab. 12. (intracerebral or intracranial adj3 hemorrhage*).ti,ab. 13. 6 or 7 or 8 or 9 or 10 or 11 or 12 14. 5 and 13 15. (animal/ or nonhuman/) not human/ 16. 14 not 15 17. limit 16 to exclude medline journals |
| **Cochrane Library (CENTRAL)** | #1 MeSH descriptor: [Lung Transplantation] explode all trees #2 (lung NEAR/3 transplant*):ti,ab,kw #3 (pulmonary NEAR/3 transplant*):ti,ab,kw #4 #1 OR #2 OR #3 #5 MeSH descriptor: [Stroke] explode all trees #6 MeSH descriptor: [Cerebrovascular Disorders] explode all trees #7 (stroke* OR cerebrovascular* OR CVA):ti,ab,kw #8 #5 OR #6 OR #7 #9 #4 AND #8 |
| **Web of Science** | TS=(("lung transplant*" OR "pulmonary transplant*") AND (stroke* OR cerebrovascular* OR "cerebrovascular accident" OR CVA OR "brain infarction" OR "cerebral infarct*")) |

*Search date: December 30, 2025. No language restrictions were applied during the search phase.*

**Supplementary Table 2. Newcastle-Ottawa Scale Quality Assessment**

The Newcastle-Ottawa Scale (NOS) was adapted for incidence studies, evaluating three domains: Selection (S1–S4, maximum 4 stars), Comparability (C1–C2, maximum 2 stars), and Outcome (O1–O3, maximum 3 stars). Total scores of 7–9 were considered high quality, 4–6 moderate quality, and 0–3 low quality.

| **Domain** | **Items** |
| --- | --- |
| **Selection (max 4 stars)** | S1: Representativeness of exposed cohort S2: Selection of non-exposed cohort S3: Ascertainment of exposure S4: Demonstration that outcome was not present at start |
| **Comparability (max 2 stars)** | C1: Control for main confounders C2: Control for additional confounders |
| **Outcome (max 3 stars)** | O1: Assessment of outcome O2: Adequate follow-up length O3: Adequacy of follow-up (≤20% loss) |

| **Study** | **Year** | **S1** | **S2** | **S3** | **S4** | **C1** | **C2** | **O1** | **O2** | **O3** | **Total (Grade)** |
| --- | --- | --- | --- | --- | --- | --- | --- | --- | --- | --- | --- |
| Shou | 2023 | ★ | ★ | ★ | ★ | ★ | ★ | ★ | ★ | ★ | 9 (High) |
| Shigemura | 2013 | ★ | ★ | ★ | ★ | – | – | ★ | – | ★ | 6 (Moderate) |
| Chan | 2023 | – | ★ | ★ | ★ | – | – | ★ | ★ | ★ | 6 (Moderate) |
| Zanotti | 2014 | – | ★ | ★ | ★ | ★ | – | ★ | ★ | ★ | 7 (High) |
| Marin-Diez | 2021 | ★ | ★ | ★ | ★ | – | – | ★ | ★ | ★ | 7 (High) |
| Makey | 2018 | – | ★ | ★ | ★ | – | – | ★ | ★ | ★ | 6 (Moderate) |
| Siddiqui | 2024 | – | ★ | ★ | ★ | – | – | ★ | ★ | ★ | 6 (Moderate) |
| Moneke | 2023 | ★ | ★ | ★ | ★ | ★ | ★ | ★ | ★ | ★ | 9 (High) |
| Smith | 2006 | ★ | ★ | ★ | ★ | – | – | ★ | ★ | ★ | 7 (High) |
| Kalsbeek | 2022 | ★ | ★ | ★ | ★ | – | – | ★ | ★ | ★ | 7 (High) |
| Akagi | 2017 | – | ★ | ★ | ★ | ★ | – | ★ | ★ | ★ | 7 (High) |
| Mohite | 2014 | ★ | ★ | ★ | ★ | ★ | ★ | ★ | ★ | ★ | 9 (High) |
| Bates | 2017 | ★ | ★ | ★ | ★ | – | – | ★ | ★ | – | 6 (Moderate) |
| Smith PJ | 2018 | ★ | ★ | ★ | ★ | ★ | ★ | ★ | ★ | ★ | 9 (High) |
| Orlitova | 2023 | – | ★ | ★ | ★ | – | – | ★ | ★ | ★ | 6 (Moderate) |
| Lusebrink | 2024 | ★ | ★ | ★ | ★ | ★ | ★ | ★ | – | ★ | 8 (High) |
| Gamez | 2017 | ★ | ★ | ★ | ★ | ★ | ★ | ★ | ★ | ★ | 9 (High) |

**Summary:** 11 studies (64.7%) rated high quality (≥7 stars); 6 studies (35.3%) rated moderate quality (4–6 stars); no study rated low quality. ★ indicates criterion met; – indicates criterion not met or not applicable.

**Detailed Quality Notes**

| **Study** | **Key Quality Considerations** |
| --- | --- |
| **Shou 2023** | UNOS registry with standardized coding; multivariable analysis with 20+ covariates; limited to in-hospital events |
| **Shigemura 2013** | Short time window (2 weeks); no stroke-specific risk factor analysis |
| **Chan 2023** | Bilateral transplant only with multiple exclusion criteria; limited generalizability |
| **Zanotti 2014** | Selected cohort (coronary angiography patients); multivariable adjustment available |
| **Marin-Diez 2021** | Imaging confirmation for all cases; may miss asymptomatic events |
| **Makey 2018** | Selected cohort (CAD risk screening); stroke as secondary outcome |
| **Siddiqui 2024** | Subgroup with anemia only; stroke as component of composite outcome |
| **Moneke 2023** | Comprehensive multivariable analysis; clear time windows (≤1 month, 2–12 months) |
| **Smith 2006** | Complete cohort with UNOS comparison; limited stroke definition detail |
| **Kalsbeek 2022** | STS definition + imaging confirmation; insufficient events for multivariable analysis |
| **Akagi 2017** | PAH patients only; limited generalizability to overall LTx population |
| **Mohite 2014** | Zero-event study; prospective complication database |
| **Bates 2017** | >20% loss to follow-up; different surgical eras compared |
| **Smith PJ 2018** | Stroke/TIA combined; comprehensive chart review protocol |
| **Orlitova 2023** | ECLS patients only; high-risk subpopulation |
| **Lusebrink 2024** | Propensity-matched design; time window not clearly defined |
| **Gamez 2017** | ICD-10 coding; only study reporting median stroke onset time |

**Supplementary Figure Legends**

*Note: Supplementary figures are submitted as separate image files (TIFF format, 300 DPI, RGB color mode).*

**Supplementary Figure 1. Leave-one-out Sensitivity Analysis.**

Forest plot displaying the pooled stroke incidence estimate after sequentially excluding each study. Point estimates remained stable across all exclusions (range: 3.44%–3.89%), confirming the robustness of the main analysis.

**Supplementary Figure 2. Forest Plot Excluding Registry Study.**

Random-effects meta-analysis of stroke incidence after excluding the UNOS registry study (Shou 2023). The pooled incidence from 16 single-center studies was 3.89% (95% CI: 3.35%–4.50%) with I² = 0%, indicating that methodological heterogeneity between registry and single-center studies contributed substantially to overall heterogeneity.

**Supplementary Figure 3. Trim-and-fill Funnel Plot.**

Funnel plot with trim-and-fill analysis for publication bias assessment. No studies were imputed by the trim-and-fill algorithm, suggesting that observed funnel plot asymmetry reflects heterogeneity in stroke ascertainment methods rather than missing studies due to publication bias.

**Supplementary Figure 4. Forest Plot Restricted to Studies with Objective Stroke Confirmation.**

Random-effects meta-analysis of stroke incidence restricted to studies with objective confirmation (imaging or ICD coding; k = 4). The pooled incidence was 4.23% (95% CI: 3.36%–5.30%) with I² = 0%, demonstrating that variation in stroke ascertainment methods is the primary driver of between-study heterogeneity.
