# Supplementary figures and images for: Stroke after lung transplantation: a systematic review and meta-analysis
Source: Front Med (Lausanne). 2026 Mar 31;13:1795510. doi: 10.3389/fmed.2026.1795510 (PMC13076320; doi:10.3389/fmed.2026.1795510)

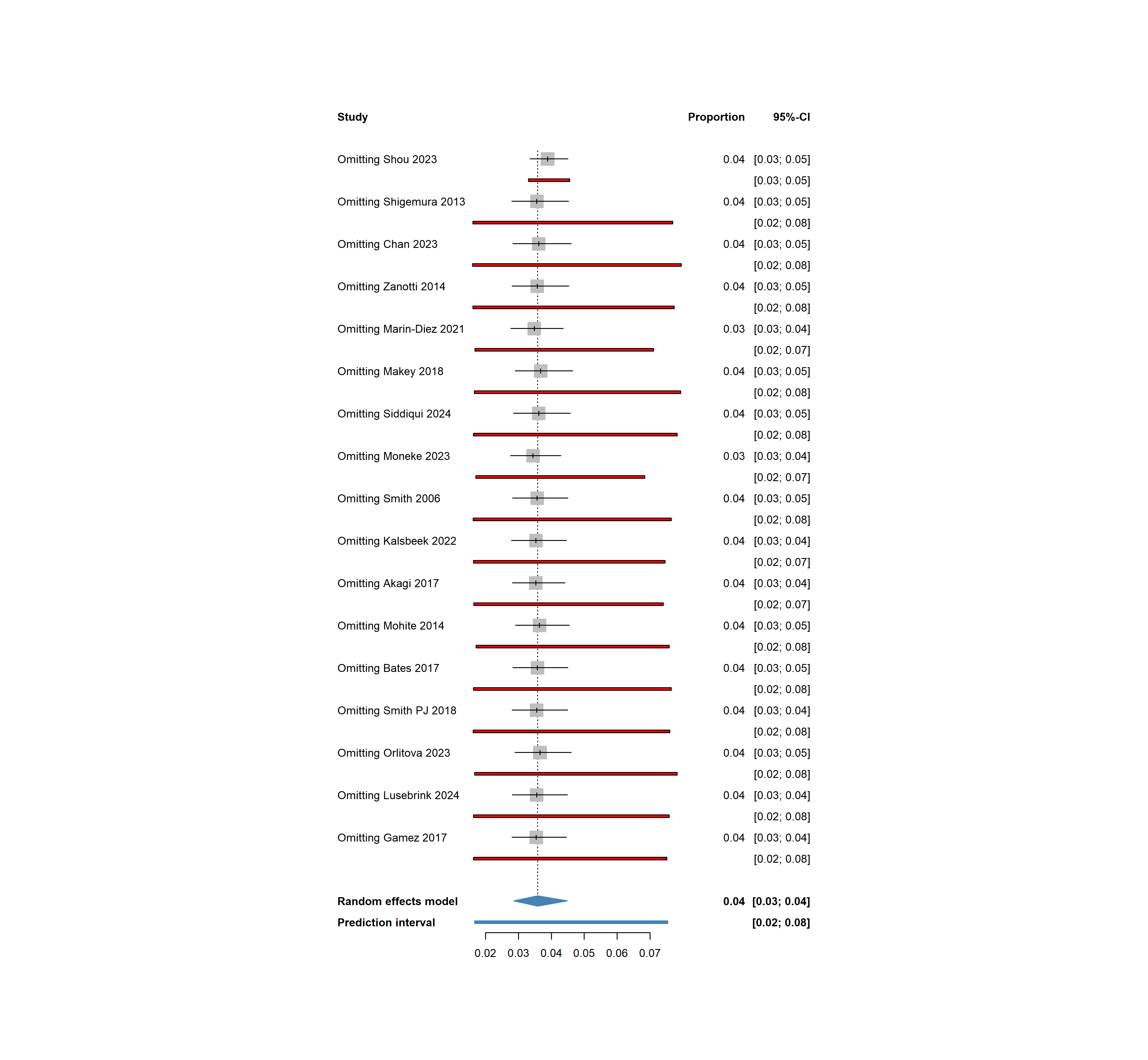

Supplement: Supplementary file 3 [file Image_1.tiff]

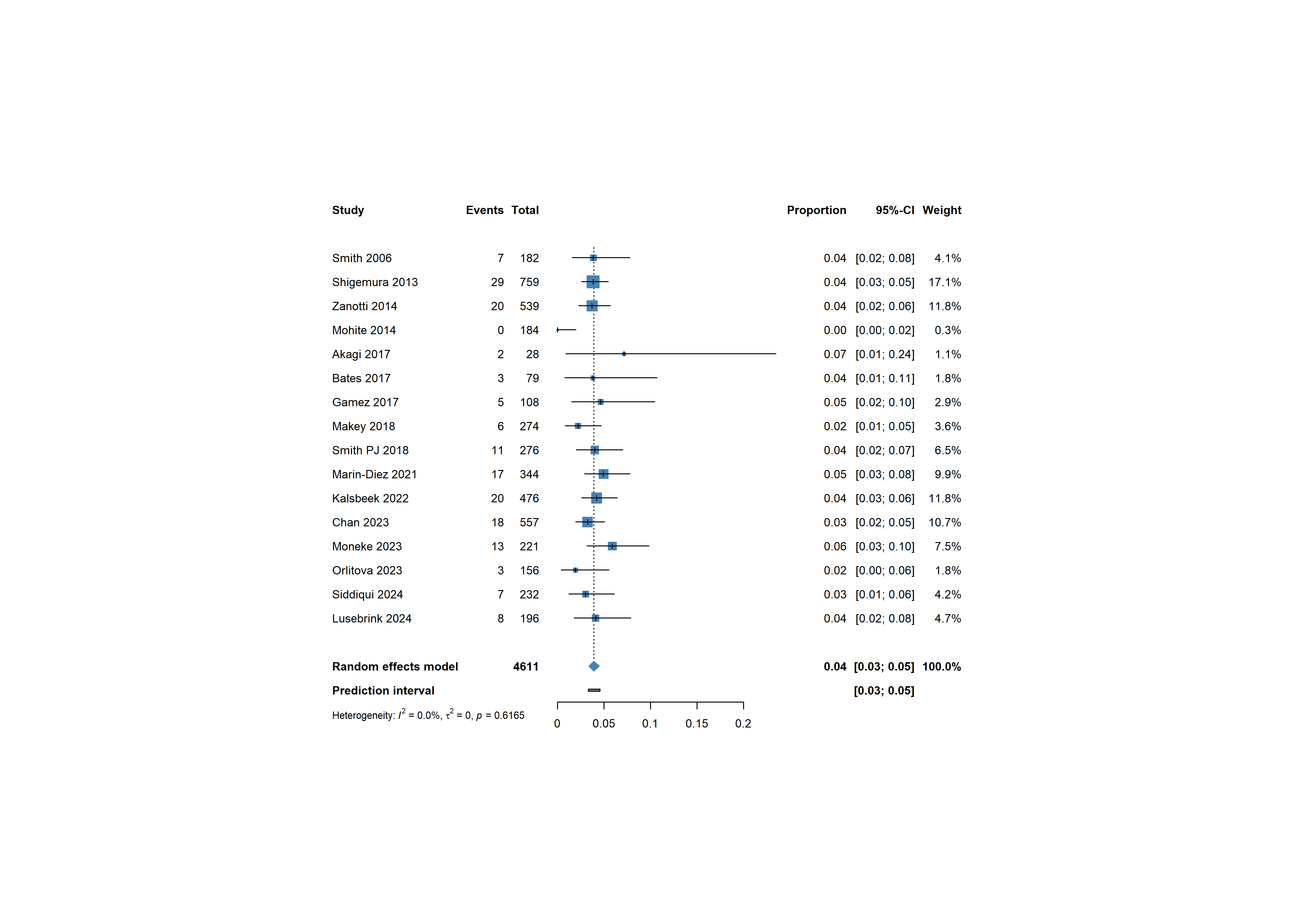

Supplement: Supplementary file 4 [file Image_2.tiff]

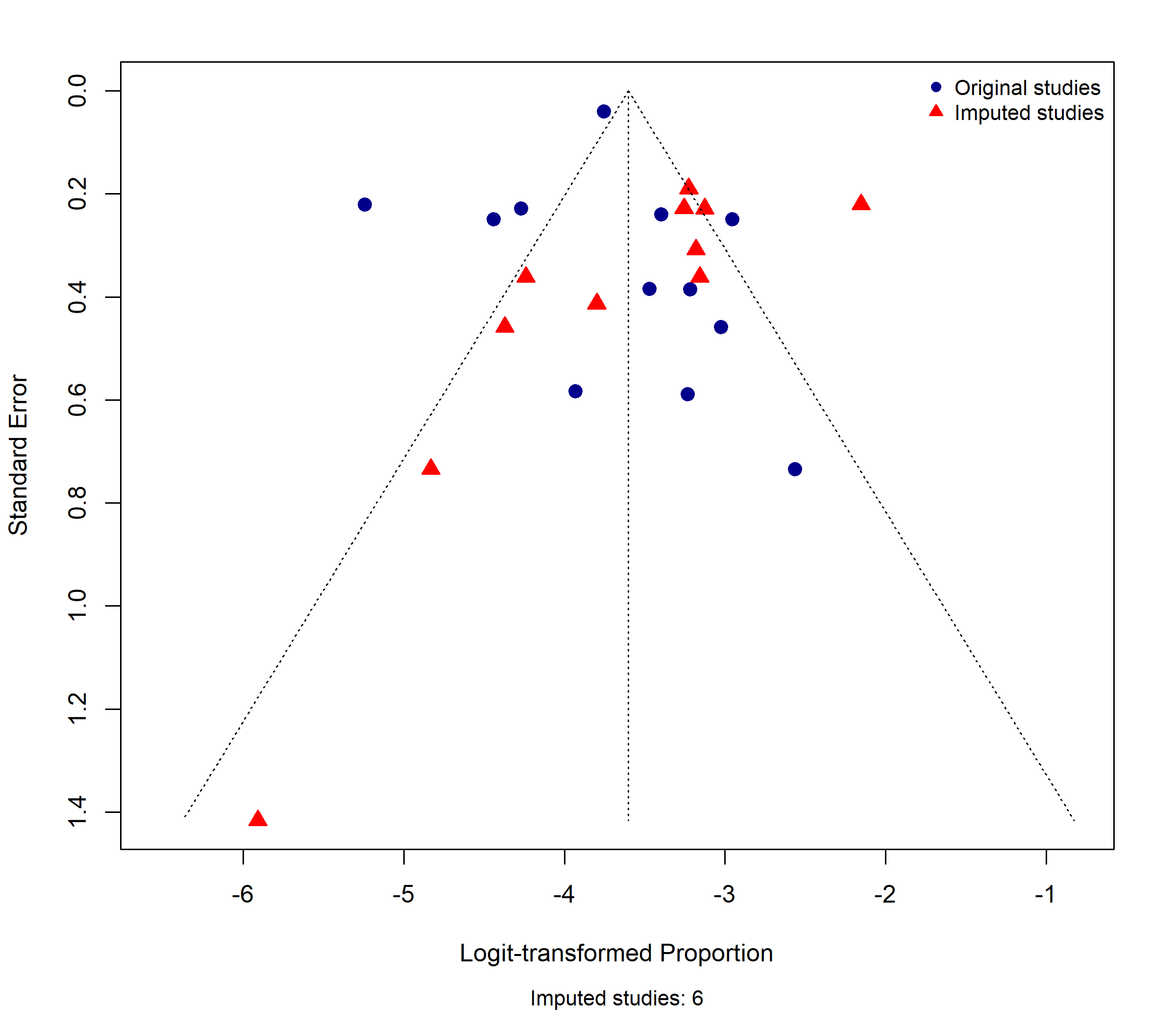

Supplement: Supplementary file 5 [file Image_3.tiff]

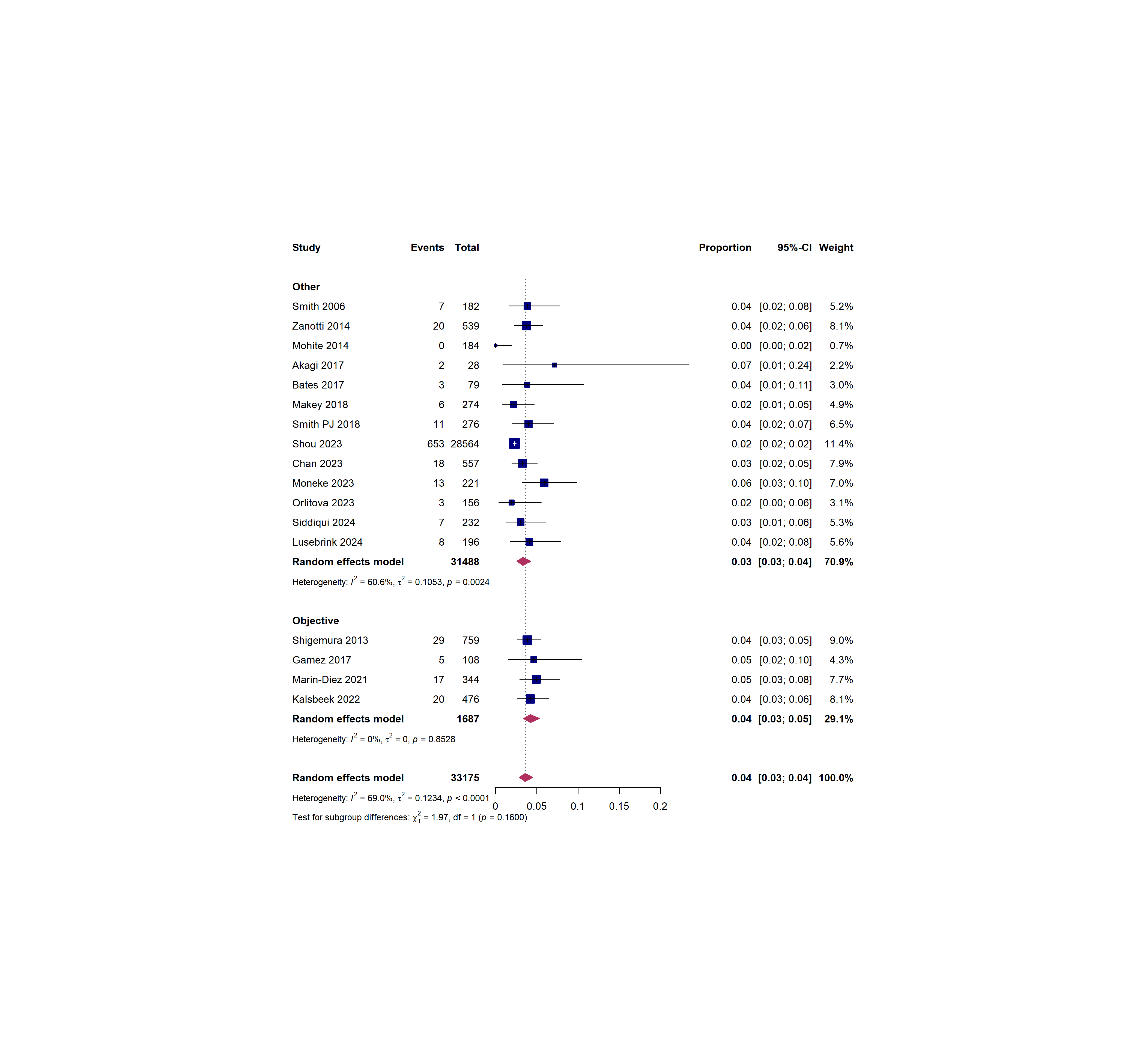

Supplement: Supplementary file 6 [file Image_4.tiff]
